# Supplementary material for: Successful Eradication of Porcine Epidemic Diarrhea in an Enzootically Infected Farm: A Two-Year Follow-Up Study
Source: Pathogens. 2021 Jul 1;10(7):830. doi: 10.3390/pathogens10070830 (PMC8308665; doi:10.3390/pathogens10070830)
Supplement: Supplementary file 1 [file pathogens-10-00830-s001.zip › pathogens-1208314-supplementary.pdf]

**Table S1.** Comparison of the full-length genome of KNU-1907 to the Korean G2b prototype KNU-141112 strain.

| Genome Region (Nucleotide Length) | KNU-1907 % Identity (No. of nt or aa Differences) to KNU-141112 |
|---|---|
| 5′-UTR (292) | 99.6 (1) |
| ORF1ab (20,345) | 99.7 (19) |
| nsp1 (363) | 99.1 (1) |
| nsp2 (2,322) | 99.8 (1) |
| nsp3 (4,863) | 99.6 (6) |
| nsp4 (1,443) | 99.3 (3) |
| nsp5 (906) | 99.6 (1) |
| nsp6 (840) | 100 (0) |
| nsp7 (249) | 100 (0) |
| nsp8 (585) | 100 (0) |
| nsp9 (324) | 100 (0) |
| nsp10 (405) | 100 (0) |
| nsp11 (54) | 100 (0) |
| nsp12 (2,781) | 99.6 (3) |
| nsp13 (1,791) | 99.8 (1) |
| nsp14 (1,551) | 99.6 (2) |
| nsp15 (1,017) | 100 (0) |
| nsp16 (906) | 99.6 (1) |
| Spike (4,161) | 99.2 (11) |
| ORF3 (675) | 99.5 (1) |
| E (231) | 100 (0) |
| M (681) | 100 (0) |
| N (1,326) | 98.8 (5) |
| 3′-UTR (334) | 99.4 (2) |
| Total | 99.5 (1/36/2)[a] |

[a] The number of individual differences in the 5′-UTR, protein-coding region, and 3′-UTR, respectively.

**Table S2.** Comparison of the full-length genome among KNU-1907 strains.

| Genome Region (Nucleotide Length) | KNU-1907-3 | KNU-1907-8 |
|---|---|---|
| | % Identity (No. of nt or aa Differences) to KNU-1907 | |
| 5′-UTR (292) | 100 (0) | 99.6 (1) |
| ORF1ab (20,345) | 99.8 (10) | 99.8 (11) |
| nsp1 (363) | 100 (0) | 100 (0) |
| nsp2 (2,322) | 99.8 (1) | 99.7 (2) |
| nsp3 (4,863) | 99.9 (1) | 99.9 (1) |
| nsp4 (1,443) | 99.5 (2) | 99.5 (2) |
| nsp5 (906) | 99.6 (1) | 99.6 (1) |
| nsp6 (840) | 100 (0) | 100 (0) |
| nsp7 (249) | 100 (0) | 100 (0) |
| nsp8 (585) | 100 (0) | 100 (0) |
| nsp9 (324) | 100 (0) | 100 (0) |
| nsp10 (405) | 100 (0) | 100 (0) |
| nsp11 (54) | 100 (0) | 100 (0) |
| nsp12 (2,781) | 99.6 (3) | 99.6 (3) |
| nsp13 (1,791) | 100 (0) | 100 (0) |
| nsp14 (1,551) | 100 (0) | 100 (0) |
| nsp15 (1,017) | 99.4 (2) | 99.4 (2) |
| nsp16 (906) | 100 (0) | 100 (0) |
| Spike (4,161) | 99.7 (4) | 99.3 (8) |
| ORF3 (675) | 99.5 (1) | 99.5 (1) |
| E (231) | 100 (0) | 100 (0) |
| M (681) | 100 (0) | 100 (0) |
| N (1,326) | 99.7 (1) | 99.3 (3) |
| 3′-UTR (334) | 100 (0) | 99.7 (1) |

| | | |
|---|---|---|
| Total | 99.9 (0/16/0)[a] | 99.8 (1/23/1)[a] |

[a] The number of individual differences in the 5′-UTR, protein-coding region, and 3′-UTR, respectively.

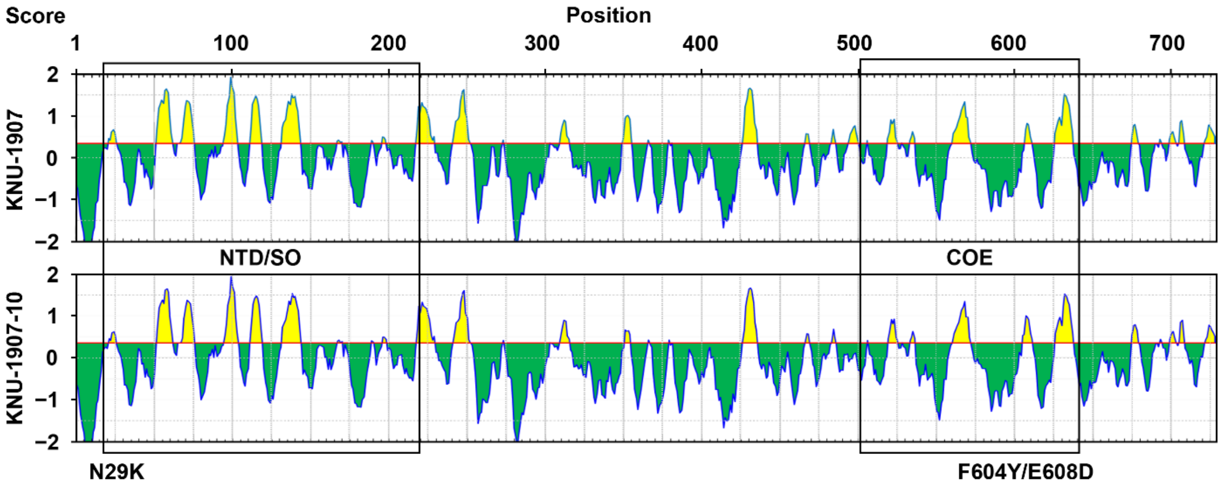


**Figure S1.** Comparison of Bepipred linear epitope prediction of the S proteins between the KNU-1907 (top) and KNU-1907-10 (bottom) strains. The regions with predicted epitopes on the protein are shown in yellow. Two NTD/SO and COE neutralizing epitope regions at positions 19-220 and 502-641, including one (N29K) and two (F604Y and E608D) amino acid changes between KNU-1907-10, respectively, are indicated by a solid box.

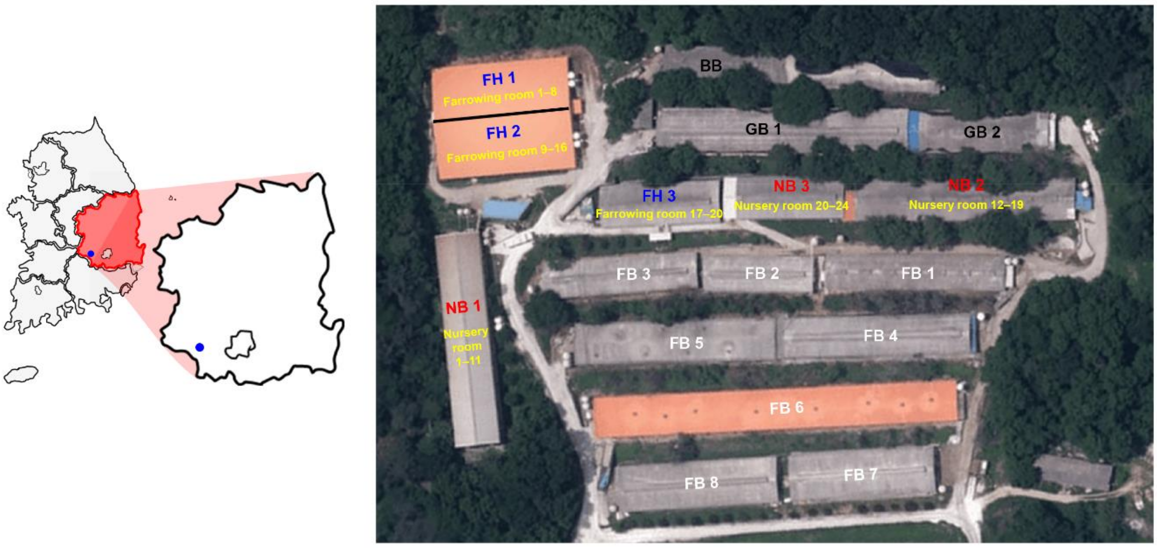


**Figure S2.** Geographic location and facility layout plan of the PEDV-infected farm analyzed in this study. A map of eight provinces in South Korea is shown on the left. The right is a magnified version of the Gyeonbuk Province in the picture on the left, and the location of the farm in this study is marked with a blue dot. The facility layout plan of the swine farm was drawn using the Google Map service (http://www.google.co.kr, accessed on 16 January 2021). The facility layout plan of the swine farm was drawn using the breeding barn (BB: black), two gestation barns (GB: black), threw farrowing houses (FH: blue), three nursery barns (NB: red), and eight finisher barns (FB: white), FH 1, 2, and 3 containing farrowing room 1–8, 9–16, and 17–20, respectively, whereas NB 1, 2, and 3 contain nursery rooms 1–11, 12–19, and 20–24, respectively.
